# Nexus between Climate-Smart Livestock Production Practices and Farmers’ Nutritional Security in Pakistan: Exploring Level, Linkages, and Determinants

**DOI:** 10.3390/ijerph19095340

**Published:** 2022-04-27

**Authors:** Pomi Shahbaz, Azhar Abbas, Babar Aziz, Bader Alhafi Alotaibi, Abou Traore

**Affiliations:** 1 Institute of Agricultural and Resource Economics, University of Agriculture Faisalabad, Faisalabad 38040, Pakistan; pomi1781@gmail.com; 2 Department of Economics, Government College University Lahore, Lahore 54000, Pakistan; babar.aziz@gcu.edu.pk; 3 Department of Agricultural Extension and Rural Society, College of Food and Agriculture Sciences, King Saud University, Riyadh 11451, Saudi Arabia; 4 Department of Community Sustainability, College of Agriculture and Natural Resources, Michigan State University, 328 Natural Resources Building, East Lansing, MI 48824, USA; traoreab@msu.edu

**Keywords:** climate-smart, livestock, food and nutrition security, Pakistan

## Abstract

Livestock plays a vital role in humans’ food and nutrition security under rapidly changing climatic scenarios. This study investigates the nature and factors affecting livestock farmers’ choices of climate-smart livestock practices by using a multivariate probit model and then estimates the average effect of these adopted strategies on per capita daily dietary (calorie, protein, and calcium) intake among livestock herders. For this purpose, data were collected from 196 livestock farmers residing in the Punjab province of Pakistan, selected through multistage purposive and random sampling. The Simpson diversity index results revealed that farmers used diversified food in their daily diet. The results also showed that farmers consumed more protein-rich food items as compared to calorie and calcium-rich food items in their daily diet. Moreover, the average per capita calorie intake of livestock farmers was 2413.19 kcal/day. Livestock farmers adopting a higher number of climate-smart livestock practices consumed more daily per capita calories, protein, and calcium compared to those who adopted a lower number of climate-smart livestock practices on livestock farms. Moreover, climate-smart livestock practices produced more and better nutritional outcomes in combination with each other than in isolation. Livestock training was found to be positively associated with the adoption of more climate-smart practices. Therefore, livestock training is necessary to expedite the adoption of climate-smart practices and to improve the nutritional security of the farmers.

## 1. Introduction

Climate change is the biggest threat to human existence, and humanity is already witnessing its impacts in the form of rising temperatures, higher frequency of erratic weather events (floods, droughts, and heat waves) and abnormal rainfalls, worldwide [1]. These climatic changes have serious consequences for low- and middle-income communities, which are more vulnerable to food and nutrition insecurity [2]. Therefore, the future impacts of climate change will be most visible among the populations of Africa and South Asia [3], where the majority of the population is poor and lives in rural areas. In addition, these changes will have consequences not only for human livelihoods but also for other social systems such as education, health and food [4]. It is evident from the fact that there are around 820 million undernourished people on the planet, a number which is growing exorbitantly as a result of international conflicts and climate change [5]. However, nations with limited adaptation capacity can hardly ensure the food and nutrition security of the masses. It is noticeable that the prevalence of severe food insecurity increased from 11% in 2017 to 14% in 2018 in South Asian countries, particularly in Pakistan [5].

Food and nutrition insecurity is frequently portrayed as a consequence of climate change. Climate change limits the ability of food systems to meet the food security and nutrition needs of an ever-increasing population [6]. Climate change may intensify such a plight with the passage of time if mitigation and adaptation strategies are not put into place. Food systems, especially agriculture—being dependent on nature—are more vulnerable to climate change than any other systems [7]. The impacts on food systems do vary globally where some countries may face more severe consequences than others [8], particularly those that rely heavily on livestock for family’s food and nutrition security [9].

Livestock is the backbone of the global food system and is a major contributor to poverty alleviation and food nutrition. Livestock provides a livelihood and food security to 1.3 billion people worldwide while its contribution to the value of agricultural production is 40% globally [5]. Similarly, more than 70% of the poor people residing in South Asia and Sub-Saharan Africa earn their livelihoods directly from livestock, thus highlighting its importance as a vehicle for economic growth in these regions [10,11]. Furthermore, livestock is also the largest consumer of land resources as 80% of cropped land is dedicated to livestock activities [5]. In South Asia, the livestock sector is recognized as a major contributor to rural households’ nutrition [12]. The demand for livestock products is expected to increase with economic growth, urbanization, and changing consumer preferences [13].

The livestock sector is badly affected by global climate change, while researchers suggest finding alternatives to livestock occupations for rural households under changing patterns of climatic variables [14] or moving to other species [15]. Climate change affects the performance of the livestock sector through direct and indirect repercussions [16]. The direct impacts include animal health and welfare [17], growth, productivity, and reproductivity [18], while indirect impacts could be in the form of changes in fodder, forage quantity and quality, and feeds [19,20]. Similarly, heat stress not only has an adverse effect on animal yield and fertility, but also causes distress and diseases leading to mortality in severe cases [21,22]. Likewise, humidity and high-speed winds also have significant impacts on milk production and the reproduction of animals [23].

Thus, considering the foregoing discussion, managing livestock farms through adaptation to erratic climate conditions is inevitable for the livelihood sustainability of rural communities. Adaptation is more important for countries such as Pakistan, where livestock has a gigantic share of around 60% of the total agricultural value-added [17]. Moreover, projections portray that livestock production in Pakistan is expected to decrease by 20–30 percent in the coming years due to increasing temperatures and heat waves [17]. The decline in livestock productivity at the farm level would potentially cause persistent poverty and food malnutrition among livestock farming communities, and climate-smart practices can play a vital role in fighting issues of poverty [24] and malnutrition.

Climate-smart livestock practices (CSLPs) are necessary to maintain and enhance livestock productivity and food nutrition among rural communities. Better weather-resistant breeds with improved fodder quality and diet supplementation can improve livestock performance and reduce potential losses. Livestock diversification, such as having different weather-resistant breeds in the herd, can maintain productivity even under extreme weather conditions [25,26]. Similarly, livestock rearing with arable farming can potentially complement each other to ensure food security and farm income [27] by promoting nutritional security [28]. Thus, the adoption of CSLPs at the farm level can assist farming households to improve their food and nutrition security.

Although numerous studies have been conducted to investigate the relationship between the adoption of climate-smart agriculture practices and the nutrition security of farming households, none of the prior studies focused on the connection between CSLPs and the nutrition security of livestock farmers. Nevertheless, [29,30,31] have explored the factors affecting the adoption of CSLPs at the farm level. More research is needed not only to explore the factors affecting the adoption of CSLPs but also to assess the nutritional impact of adopted CSLPs on farm households for developing effective approaches to improve food and nutrition security. This study bridges the gap by exploring factors affecting the adoption of CSLPs and by analyzing the impact of the adopted CSLPs on livestock farmers’ food and nutrition security.

The current study answers three key research questions: First, what CSLPs farmers are implementing to lessen the effects of climate change on their farms? Second, what socioeconomic factors influence the adoption of CSLPs on farms? Third, how does the adoption of different numbers of CSLPs affect farmers’ nutritional security?

In terms of application, this research provides a useful framework for assessing the impact of CSLPs on farmers’ nutritional security. The findings of this study will aid policymakers in their assessments related to enhancing food and nutritional security in Pakistan, as well as in other developing countries facing climatic changes. The findings, in particular, identify some of the critical CSLPs that policymakers may include in their top priorities for climate change adaptation policy to improve the food and nutrition security of rural households.

The rest of the research is organized as follows. The “Materials and Methods” section of the study describes the data collection process, questionnaire design, conceptual framework, and econometric techniques used to answer the research questions. The “Results” section comprises the findings of the study. The “Discussion” section discusses the results in the light of prior studies. “Conclusions” is the last section of the study, and it sums up important findings and policy suggestions.

## 2. Materials and Methods

### 2.1. Description of the Study Area and Data Collection Procedure

The annual minimum and maximum temperatures in Pakistan remained between 9.5 °C and 22.87 °C and 16.46 °C and 36.44 °C, respectively, during 1990–2015. During the same period, the mean annual temperature of Pakistan was 21 °C and the average annual precipitation was 301 mm [32]. Pakistan is the 5th most-affected country due to climate change globally, and the mean annual temperature of the country has risen by 1 °C during the last century. Similarly, the amount of precipitation increased by 18% to 32% in the rainy season (the monsoon season) during this period [33]. Moreover, the number of heat-wave days per year has also increased significantly during the last three decades. Moreover, Pakistan’s mean annual temperature is expected to increase by 3 °C to 6 °C by the end of the twenty-first century [34]. The increase in mean annual temperature, along with other climatic vulnerabilities such as floods, droughts, heatwaves, and precipitation change affect crop and livestock productivity [16], which amplifies the problem of food and nutritional security in the country [35].

During the period 1980–2018, the annual mean minimum and maximum temperatures in Punjab remained between 16.52 °C and 21.50 °C and 30.09 °C and 32.75 °C, respectively. The mean annual precipitation in Punjab was 532.5 mm during the same period, of which 50–75% occurred from June to August [15]. Punjab is the largest province of Pakistan and is vulnerable to climate change due to its geographical location, low adaptive capacity, and high dependence on the natural environment [36]. In 2010, Punjab faced the worst floods in its history, which forced millions of people to flee their homes, destroyed agricultural crops, and killed animals [37].

**Figure 1:**
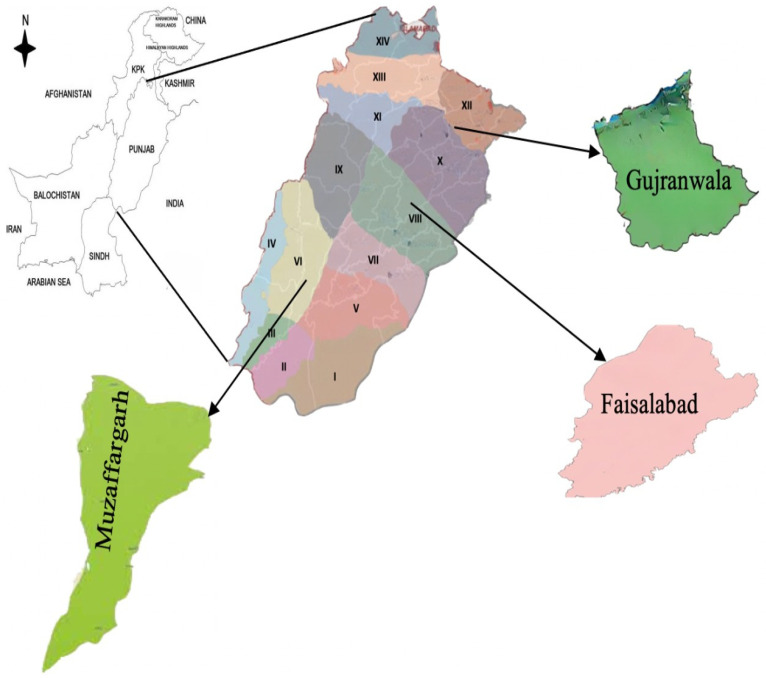
Study districts with the specification of Agro-Ecological Zones to which they belong (Roman numerals show AEZs).

On the agricultural front, the Punjab province alone contributes two-thirds of the national agricultural production. Livestock farming is a major occupation in rural areas of the province and is considered an indispensable part of rural life. The province has the most number of buffalos (16.019 million) compared with other provinces [17,38]. Keeping in view the climate vulnerabilities, herd size, and rural population (10.5 million households), the Punjab province was selected as the study area for this study (Figure 1).

Cochran’s [39] formula was used to estimate the representative sample size for this study as given below:

*n*_0_ = *Z*^2^ × *p* × *q*/*e*^2^
*n* = sample size
*Z* = abscissa of the normal curve that cuts off an area α at the tails
*e* = precision level
*p* = estimated proportion of an element
*q* = 1 − *p*

Assuming *p* = 0.5 (maximum variability), anticipated confidence interval, ±7% precision level, and *Z* value of 1.96, 196 rural livestock households were extracted as the total sample size for this research. The calculated sample size was distributed to the smallest administrative units of villages by using multistage purposive and random sampling techniques.

**Figure 2:**
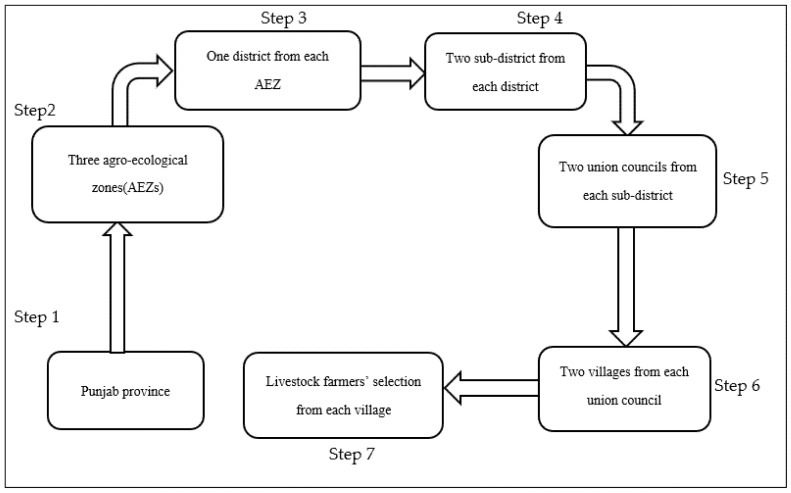
Step by step sampling procedure.

The Punjab province is divided into different agro-ecological zones (AEZ) based on climate characteristics, crop norms, and soil features [40]. In the second step of multistage sampling, three AEZs (mixed cropping zone, maize-wheat mix zone, and the rice zone) with the largest animal inventory were selected from the Punjab province. The mixed cropping zone (zone VI) receives 460 mm of rain annually, and its mean minimum and mean maximum temperatures vary between 13 °C and 40 °C, respectively. The maize-wheat mix zone (Zone VIII) receives 590 mm of rain annually, and its mean minimum and mean maximum temperatures vary between 11 °C and 38 °C annually. The third selected zone is the rice zone (zone XII), with 1250 mm of annual rainfall and a 12 °C to 36 °C mean minimum and mean maximum temperature annually [40]. As each AEZ consists of several districts, in the third step of sampling, one district with the highest number of buffalo and cattle together (Muzaffargarh 1.79 million, Faisalabad 1.11 million, and Gujranwala 0.82 million [17]) was selected from each AEZ. As each district consists of three to five sub-districts (tehsils), we randomly selected two sub-districts from each district in the fourth stage. In the fifth step of sampling, two union councils (smaller administrative unit comprising 4–8 villages) from each tehsil were randomly selected. In the sixth step, two villages from each union council were selected. In the seventh and final step of sampling, eight to nine livestock farmers were selected randomly for interviews from the households’ lists. The step-by-step procedure of sampling is shown in Figure 2.

### 2.2. Brief Literature Review and Conceptual Model

Climate-smart practices aim to increase farm productivity and income with minimum environmental impacts, thus indirectly improving the food and nutrition security of households [41]. Farmers who are well aware of the impacts of climate change on their farm productivity respond to them by taking different measures at the farm level. Many studies have triumphed to cover various aspects of climate change, livestock farming and the adoption of CSLPs for the sustainability of rural incomes and to ensure food security.

In this regard, Issahaku et al. [42] analyzed the nexus of climate-smart practices and farmers’ food and nutrition security in Ghana. This study reported that the adoption of different climate-smart practices at the farm level positively influences the food and nutrition security of farming households. Similarly, [43,44] examined the effect of the adoption of climate-smart practices on farmers’ nutrition security in the Punjab/Pakistan but with little focus on the factors of such uptake. They noted a weak linkage between the adoption of climate-smart practices and the nutritional security of farming households. Shahzad and Abdulai [44] further noted that the adoption of climate-smart practices does not occur uniformly at all farms due to differences in adaptive capacity and socioeconomic characteristics.

Abegunde et al. [45] analyzed the factors affecting the adoption of climate-smart practices in South Africa and revealed that schooling years, farming experience, total farmland, media access, and organizational membership influence the climate change adaptation strategies among farmers. Aryal et al. [46] analyzed the factors affecting the adoption of climate practices in the Bihar state of India and found that educational status, social and economic capital; farmland, market accessibility, and access to training determine the adoption status of different climate-smart measures at farms. In a similar vein, Sardar et al. [47] conducted a study on determinants of climate change adaptation practices and the implications of adopted strategies on farm income in the Punjab province of Pakistan and reported that institutional factors, financial resources, size of farmland, and education level of the farmers affect the adoption of different climate practices at the farm level. Shahbaz et al. [48] also conducted a study on the determinants of climate practices in the Punjab province of Pakistan and reported that education, farming experience, livestock training, and working hours significantly affect the adoption of CSLPs on farms. Faisal et al. [49] analyzed the risk perception and factors influencing the adoption of climate-smart practices at livestock farms in Pakistan and revealed that literacy rate, livestock experience, and institutional services (veterinary hospital, veterinary doctors, and livestock training) affect the adoption of various climate change practices at the farm level.

**Figure 3:**
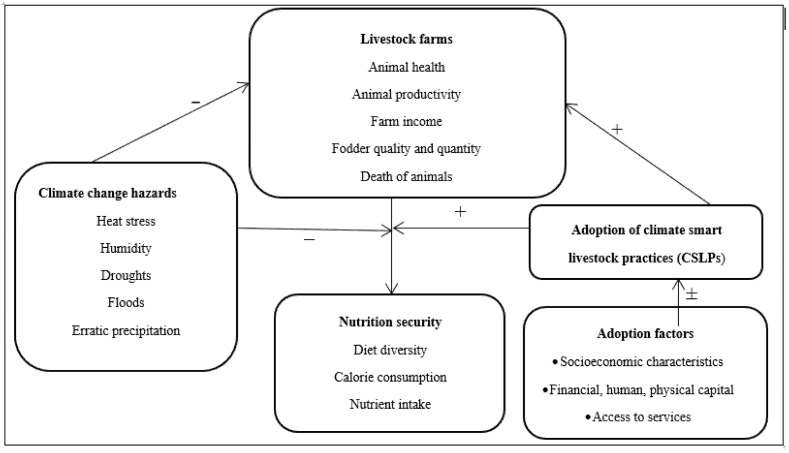
Conceptual model of the study.

This work has taken insights from the literature cited above and in the introduction section to develop the conceptual framework for this study in order to build a nexus between CSLPs and nutrition security, the level of uptake of CSLPs and its determinants (Figure 3). It is evident from Figure 3 that climate change negatively affects livestock productivity which ultimately affects the nutritional security of the livestock farmers. The livestock farmers are obliged to take appropriate measures at the farm level to counter these effects which we term as CSLPs. The adopted CSLPs minimize the effects of climate change on animal health, productivity, and farm income while at the same time reducing livestock morbidity and mortality, thus assisting farmers to maintain and improve their nutrition security. However, the adoption of CSLPs at the farm level is constrained by a range of factors related to the farm, market, institutions, household and personal characteristics of the farmers.

### 2.3. Questionnaire Design

A well-structured questionnaire was used to collect data through face-to-face interviews with the selected livestock farmers. The questionnaire was organized into different modules. The first part of the questionnaire contained the demographic characteristics of farmers such as human capital, financial and physical capital, and access to services. The second part of the questionnaire comprised queries related to climate-smart livestock practices (diversification, feed, and weather-resistance options). Each of the three CSLPs entailed further sub-components of practices. The diversification strategy included livestock diversification, different breeds of the same animals, and mixed farming as a strategy to counter climate change. The second strategy of CSLP had components related to feed usage, such as the use of silage, drought-tolerant fodder varieties, traditional supplements (salt, mustard oil, jaggery), and feed concentrates. The weather-resistance strategy included shelter or farm management, improved drainage, using trees for shade, using fans, water collection and storage, and brick husk usage as strategies to minimize climate effects.

The third part of the questionnaire was about the consumption of food items by livestock farmers. Food data about 42 food items for 12 months was sought for this purpose. This part was further divided into sub-components according to the availability of food items. Some of the food items (vegetables and fruits) are available only during a particular season, and therefore, they were kept in a separate section according to their availability. For food items available year-round (such as eggs, meat, milk, etc.), a separate sub-section was dedicated in the questionnaire.

A pretesting of the questionnaire was carried out before the final survey in order to redesign the questionnaire in case some information of particular significance skipped out, such as the adaptation option. This is because adaptation is a local subject, and strategies in one region may not be implementable in other regions. A team of five members, comprising of experienced male and female enumerators, collected the final data. Two days of training were provided to the data collection team to obtain precise data from the livestock farmers, especially related to food quantities. The selected farmers were contacted before visiting them for data collection and informed consent was accorded before the formal interview. The information on food consumption was also validated from their monthly records (where available) in order to obtain precise food consumption data.

### 2.4. Data Description

#### 2.4.1. Household Dietary Intake and Food Diversity Determination

The indicators employed to assess the nutrition status of livestock farmers in this study include per adult nutrient intake of calories, protein, and calcium. In addition to this, the Simpson index was used to measure diet. The Simpson index is used as a proxy to measure nutrition adequacy and shows households’ access to diversified food items [50]. Diversity in the diet is a vital component of nutrition security, and the intake of a variety of food items from different food groups ensures essential nutrients [51]. If a household consumes food items from different food groups in unequal proportions, it will have less dietary diversity than a household that consumes food items from different food groups in equal proportions. The food items in Pakistan are grouped into the following categories: (i) cereals, (ii) vegetables, (iii) fruits, (iv) milk and milk products, (v) meat and pulses, and (vi) fats and oils [2]. We used these six food groups to compute dietary diversity for calories, protein, and calcium usage with the following formula:

$$D=1-\overset{n}{\underset{f=1}{\sum }}p_{i}^{2}$$

where:

*D* = Dietary diversity
*p_i_* = Share of food items in total dietary intakes consumed (calories, protein, and calcium)
*n* = food group
*f* = 1, ……, 6

The value of dietary diversity ranges between 0 and 1. A value close to 0 indicates less diversity and a value close to 1 shows high diversity in dietary intakes. Apart from measuring the Simpson diversity index for dietary intakes, we also determined the average daily intakes for each of the nutrition indicators from the collected data. The quantities consumed for each food item were converted into calories, protein, and calcium by using the food composite table index prepared by the Food and Agriculture Organization and the Government of Pakistan jointly [52].

#### 2.4.2. Components of CSLPs

A sustainable food system is defined as a system that provides food security and nutrition to the present generation without compromising the social, economic, and environmental aspects of future generations [5]. A number of climate-smart livestock practices being used in the study area were considered to be components of the three broad categories, namely diversification (D), feeding strategies (F), and weather-resistance strategies (W). Diversification is a commonly adopted strategy by livestock farmers to minimize the effects of climate change. This broad category included practices such as livestock diversification, having multiple breeds of the same animal species, and mixed farming. Livestock diversification means having different animal species such as cows, buffaloes, and goats simultaneously on the farm. Having different breeds of the same animal, such as buffaloes, was also considered to be a component of diversification. Both of these components were measured qualitatively (yes or no). Similarly, mixed farming was considered as cultivating crops along with animals.

Feeding strategies, both conventional (salt, jaggery, mustard oil, seed cotton, and wheat bran) and unconventional (drought-resistant fodder and forage varieties), are being implemented at livestock farms in the study area. For some months in Pakistan (April–June), green fodder is scarce due to high temperatures; farmers use silage or wheat straw as a feeding strategy for livestock farms during these months.

The weather-resistance category (W) includes the strategies used to minimize the effects of climate change, such as erratic rainfall and severe weather (heat stress and heatwaves). Farm or shelter management is a strategy adopted by livestock farmers mostly to address heat stress, heatwaves, humidity, and unpredictable rains. The other widely adopted strategy to address heat stress is the use of trees for shade. During the rainy season, livestock farmers have enough financial resources, and use proper drainage systems made of cement and bricks (local tiles) for better sewerage of rainwater on their farms whereas farmers having low financial capacity use brick husks or sand to make animals comfortable. Fans and heaters are also used to counter the effects of climate change on livestock farms. Similarly, some farmers do store rainwater to overcome water shortages in selected months. Table 1 shows eight different combinations of these practices.

Livestock farmers had the choice to select more than one combination due to different requirements in different seasons. Qualitatively, all components of each category were measured. A livestock farmer adopting at least one component from any broad category (diversification, feed, and weather resistance) was considered an adopter of that strategy. A livestock farmer adopting all three practices (diversification, feed, and weather resistance) is denoted by D1F1W1. Similarly, a livestock farmer adopting only diversification and feeding strategies at his farm is denoted by D1F1W0. About 35% of the total livestock farmers used a combination of all three practices jointly, while 27% to 43% of them adopted combinations of two practices, and 9% to 13% used a single CSLPs on their farms. There was no farmer in the sample who did not adopt a single strategy.

#### 2.4.3. Selection of Explanatory Variables

As envisioned in the conceptual framework, the adoption of CSLPs is influenced by a variety of demographic factors (age, education, gender headship), human capital (labor force, livestock experience), financial and physical capital (total landholding, animal inventory, off-farm income, credit access), and access to services (market, training, and media) [42,43,44,45,46,47,48,49,53] as shown in Table 2. Demographic characteristics such as the age of the family head and family size are important characteristics of rural farm households, as both of them can potentially (positively/negatively) affect the adoption of CSLPs. More importantly, it is the head of the family who makes all decisions to adopt or not adopt any climate change strategy.

Human capital, such as education of the household head and labor force in livestock, are also important determinants of adoption at the farm level. Educated people are expected to be more aware of climate change and its repercussions than uneducated people. Most of the strategies employed at livestock farms are laborious and conventional, and hence, need extra labor for adoption at the farm level. This variable is expected to have a positive influence on adoption. The other important requirement for adoption is the inventory of financial and physical capital. Therefore, all explanatory variables included under the financial and physical capital category are expected to relate positively to the adoption of CSLPs. Access to services such as veterinary doctors and hospitals is expected to contribute positively to adoption on livestock farms. As climate change is a pressing issue in today’s world, the government is creating awareness through the media and conducting training to guide livestock farmers to adapt to climate change. Therefore, these variables are also expected to contribute positively to the adoption of CSLPs among livestock farmers. The mean values of the explanatory factors of various CSLP adopter groups were compared using a post-hoc test.

### 2.5. Empirical Models

A two-step course of action was developed to look at the choice of different combinations of CSLPs by livestock farmers and then the adoption effects of these adopted CSLPs on dietary intake were measured through propensity score matching (PSM).

The simultaneous nature of three combinations of CSLPs (diversification practices, feeding practices, and weather-resistance practices) resulted in eight different combinations (Table 1). As livestock farmers have the choice to select one or more combinations of CSLPs at the same time, this situation favors the use of the multivariate probit model, which is specially designed to take into account the simultaneous nature of two or more than two dependent variables. The choice of the selected CSLP combination may vary from farm to farm and household to household as the ability, skills, resources, and other farm characteristics of livestock farmers are different from each other. The outcome of CSLPs can be converted into utility functions. Consider a livestock risk-averse farmer who faces the choice of whether to adopt a certain combination of CSLPs or not. Let *Uj* be the utility of adopting the combination of CSLPs. The livestock farmer will adopt *j*th combination of CSLP, if and only if its utility is greater than any other CSLP *k*th combination. Where *j* = 1, ……, 7 and *j* ≠ *k*. The utility, which the livestock farmer gets from adopting a *j*th combination, is determined by the farmer’s demographic characteristics, human capital, financial and physical capital, and access to the services as shown below:

(1) $$U_{ij}^{*}=X_{i}\beta_{j}+e_{ij}$$

where

$U_{ij}^{*}$ = Utility of adopting *j*th combination (latent variable)
*X_i_* = Livestock farmer characteristics
$\beta_{j}=\mathrm{Coeficients}$
$e_{ij}=\mathrm{Error}\;\mathrm{term}$

From Equation (1), for each combination, we can convert unobserved preferences into an observed binary outcome equation.

(2) $$I_{ij}=\{\begin{array}{c} 1,|If\;U_{ij}>0\\ 0,|Otherwise\; \end{array}\;j=1,\ldots \ldots ,7$$

The efficient adoption of CSLPs can improve livestock farmers’ productivity and can add to their per capita nutrition intake. However, it is difficult to differentiate between the nutritional outcomes of adopters and non-adopters in observatory research. In investigational research, where data is gathered through randomization and information is noted about counterfactual outcomes, it is easy to differentiate between adopters and non-adopters. In observatory research, like in this study, researchers have no control over the adoption of CSLPs, and it depends solely on the outcome. Therefore, the biased estimation of the average treatment effect is more likely for the population. It is necessary to address the issue of selection bias to estimate the unbiased net effect of the adoption of CSLP combinations on dietary intake (calorie, protein, and calcium). After Multivariate Probit (MVP) results, we estimated the inverse miller ratio to be used as an extra independent variable to address the individual heterogeneity of the underlying selection bias. Equation (3) is re-specified as shown below:

(3) $$\;Y_{ij}=\gamma X_{ij}+\alpha_{j}\tau_{ij}+\mu_{ij\;\;}\;\mathrm{If}\;Ii=j\;\mathrm{for}\;j=1,\;\ldots \ldots ,j$$

$Y_{ij}=$ Outcome vector for each of the dietary intake for *j*th combination of CSLPs
*µ_ij_* = Error term for adopting *j*th combination by *i*th farmer
*X_ij_* = Explanatory variables for jth combination for *i*th farmer
γ = Parameters of explanatory variables
$\alpha_{j}$= Correlation between error terms $e_{ij}$ and $\mu_{ij}$

#### Estimation of Average Adoption Effects

The impact of the adoption of different CSLPs on food and nutritional security was measured by using the propensity score matching approach (PSM). The PSM technique couples the treatment (adopters) and control (non-adopters) categories by considering the similarity in their apparent characteristics. PSM can be a better choice when instruments are weak or not available [54]. We used the near-neighbor method (NNM) to match the nutritional outcomes of adopters and non-adopters for different CSLPs combinations. The advantage of using the NNM approach is that it only compares units available within the caliper. Therefore, the NNM approach permits the usage of additional (fewer) units when decent matches are (not) accessible.

The most relevant estimate from PSM is the average treatment effect on adopters (ATT) and non-adopters (ATU). The average treatment effect on adopters of a certain combination (ATT) and the average treatment effect on non-adopters (ATU) retorts the query on how the average effect would have been altered if each of the farmers who implemented a specific combination of CSLPs had instead resorted to another combination of CSLPs. Similarly, the ATT answers the question of how the average effect would have changed if a farmer had implemented a set of a combination with more CSLPs instead of a combination with less CSLPs. However, ATT is generally used to attain an unbiased estimation of the average effect of a combination of more strategies. Therefore, ATT is used to compute probable nutrition outcomes for the adoption of a combination with higher CSLPs with a counterfactual nutrition assessment of a combination with fewer CSLPs. The equation below shows the factual average nutrition intake under the adoption of a combination with a higher number of CSLPs.

(4) $$E(Y_{ij}|I_{i}=j)=\gamma_{j}\;X_{ij}+\alpha_{j}\tau_{ij}\;$$

The counterfactual average nutrition intake, if the livestock farmer had adopted a combination of fewer CSLPs instead of a combination with a higher number of CSLPs, is as follows:

(5) $$\;E(Y_{im}|I_{i}=j)=\gamma_{m}\;X_{ij}+\alpha_{m}\tau_{ij}\;$$

Equation (4) represents the factual nutrition outcome of livestock farmers who adopted a combination with a higher number of CSLPs, while Equation (5) shows the counterfactual nutrition outcome for a combination with a lower number of CSLPs. These equations are utilized in order to have unbiased outcomes of the effects of the adoption of combinations of CSLP.

The average adoption effect of CSLPs is conditional on the adoption of a higher number of CSLPs and is estimated as a difference between Equations (4) and (5)

(6) $$\;ATT=E(Y_{ij}|I_{i}=j)-E(Y_{im}|I_{i}=j)=X_{ij}\left(\gamma_{j}-\gamma_{m}\right)+\tau_{ij}\;\left(\alpha_{j}-\alpha_{m}\right)\;$$

The primary goal of propensity score matching is to stabilize the assessed distribution of covariates across both adopter and non-adopter groups [55]. If after matching, there are still some unobserved variables that affect the decision of adoption and outcome variables, a hidden bias problem may persist, and thus, matched estimations will not be representative of the population [56]. Therefore, after matching, a balancing test was performed to ensure that the differences among covariates of CSLP adopters and non-adopters have been eradicated.

## 3. Results

### 3.1. Characteristics of Different CSLP Adopter Groups

The livestock farmers adopting all three strategies (D1F1W1) were found to be younger and had smaller family sizes compared to other CSLP adopter groups, but these differences are not statistically significant. The average age of livestock farmers adopting all three strategies was 47.03 years (Table 3). In addition, livestock farmers who used all three strategies had less experience in livestock rearing and fewer animals than other CSLP adopter groups. Farmers adopting only one CSLP at their farms had significantly lower access to credit facilities compared to other adopter groups.

Single strategy adopters were found to have the highest experience compared with other groups. The number of family members among the farmers simultaneously adopting all strategies was relatively higher who attended training/workshops as compared to any other group. However, the overall participation of livestock farmers in these trainings remained low in the study area. 

The overall average family size of livestock farmers was 8.19 persons. Males headed the vast majority of households. The average number of years of schooling was 7.90 among livestock farmers. The average labor force involved in livestock activities at the sampled farms was 1.83 persons. All livestock farmers had a great deal of livestock experience in farming. The average landholding size was found to be only 6.17 acres. More than half of the total livestock farmers had off-farm income sources. The average herd size was 5.45 livestock units while around two-thirds of the total livestock farmers did not have access to credit facilities.

### 3.2. Households’ Dietary Intake and Food Diversity

The per capita calorie consumption among respondent households was 2413.19, whereas protein intake remained 69.9 gm/day being higher than the required (56 gm/day) intake for a sedentary man. The average calcium intake was 657.70 mg/day (Table 4). In addition, more than 33% of the sampled farmers consumed less than the average per capita intake in the study area.

The Simpson index values indicated that study respondents consumed diversified food items from all six food groups although with a greater proportion of protein-rich foods. The Simpson food diversity index, based on protein share in daily food consumption, was estimated to be 0.70 (see Table 4).

### 3.3. Factors Affecting the Choice of CSLPs

#### 3.3.1. Demographic Factors

Results of the MVP show that the age of livestock farmers was negatively associated with the adoption of all three combinations of two practices at the livestock farm level. This indicates that older farmers are less likely to adopt a two-strategy combination. Similarly, age was also negatively associated with the adoption of weather-resistance practices in isolation (Table 5). Family size also showed a negative association with the adoption of diversification, either solitary or in combination with other practices. The household head’s gender was positively associated with the adoption of combinations involving three practices jointly. The results showed that male-headed households were more likely to adopt all three practices together on their farms. Likewise, the household head’s gender was also positively associated with two other combinations involving feed strategy, except D1F1W0.

#### 3.3.2. Human Capital

Education was positively associated with all combinations involving two or more climate-smart strategies in a combination jointly. An interesting result about the factors affecting the adoption of different climate-smart practices was the negative association of livestock experience with adoption. A livestock farmer with more experience is less likely to adopt all three practices (diversification, feed, and weather-resistance strategies) together. Having a university graduate in the family also increases the chances of adoption of all three practices together at the livestock farm.

#### 3.3.3. Physical and Financial Capital

Land ownership showed a positive link with all combinations involving two or three CSLPs. Farmers with greater landholdings were more likely to adopt those combinations involving two or three CSLPs compared with those having less landholdings. Herd size/animal inventory also showed a positive linkage with the adoption of all combinations involving diversification at sampled farms, either in combination with other practices or in isolation. Therefore, having more animals in the herd increases the chances of diversified adoption strategies along with other practices. Furthermore, the availability of credit increases the chances of adoption of those combinations involving feed strategies, whether in isolation or combined with other practices.

#### 3.3.4. Access to Services

The involvement of livestock farmers in training programs/workshops was positively associated with the adoption of CSLP combinations involving two or more climate-smart measures. Having membership in a farmers’ organization also positively affected the adoption of the CSLP combination involving all three strategies jointly. Media use for weather information was also positively associated with the adoption of all three practices jointly. Farmers having livestock training, farmer organizations’ membership, and media access were more likely to adopt three CSLPs jointly on farms compared with those having no access to such services.

### 3.4. Adoption effects of CSLPs on Daily Dietary Intake

#### 3.4.1. Adoption Effect of CSLPs on Daily Calorie Intake

Table 6 presents the conditional average effects of adopting different combinations of a diversification strategy, feeding strategy, and weather-resistance strategy on per capita calorie consumption. Each computed effect is described both in combined and in individual form. By estimating combined effects, it is possible to draw a comparison between livestock farmers who adopted a combination of a higher number of strategies (treated) and those who adopted a combination with a lower number of CSLP (untreated) for all-selected livestock farmers. The average effect of adopting a higher number of CSLPs is presented as a difference between factual and counterfactual per capita calorie consumption for each of the combinations. Rows a, b, and c compare the adoption effects of a combination having three practices with a combination having two practices. Similarly, rows c, d, and f compare the adoption effect on per capita calorie consumption of adopting a combination of three or two practices at a livestock farm with the adoption of a single practice. The difference in per capita calorie consumption of livestock farmers adopting three practices as compared to a single practice is greater than the difference between livestock farmers adopting two practices as compared to a single practice. For example, the difference between the per capita calorie consumption of a livestock farmer adopting all three practices (D1F1W1) and a single adopter (D1F0W0) is 667.82 kcal, which is greater than the difference (443.90 kcal) between an adopter of two practices (D1F1W0) and a single adopter (D1F0W0) and the difference (337.89 kcal) between an adopter of two practices (D1F0W0).

Another important conclusion can be drawn about feed and weather-resistance strategies that produce better per capita calorie intake results compared with diversification when they are used in isolation. It is because the difference (237.48 kcal) between a combination of two practices (D1F0W1) and feed practice in isolation (D0F1W0) is smaller than the difference (337.89 kcal) of the same strategies (D1F0W1) with diversification in isolation (D1F0W0). However, diversification results in better per capita calorie intake when it is combined with feed or weather-resistance practices evinced by per capita calorie difference between an adopter of three practices (D1F1W1) and partial combinations involving diversification (D1F1W0) and (D1F0W1) is less than the difference of a partial combination not involving diversification (D0F1W1). These results also depict important implications of climate-smart livestock practices: CSLPs produce better per capita calorie results when they are used in combination rather than in isolation.

#### 3.4.2. Adoption Effect of CSLPs on Daily Protein Intake

Table 7 describes the average conditional effect of the adoption of different climate-smart livestock practices on the per capita protein consumption among livestock farmers. Livestock farmers adopting all three practices (diversification, feed, and weather-resistance strategies) consumed a higher level of protein as compared to those who adopted one or two strategies. Therefore, the gains by moving from the adoption of a single climate-smart strategy to jointly adopting three strategies at a time are higher. A livestock farmer will gain 21.689 gm/day of protein per capita by shifting from the adoption of feed and weather-resistance strategies (D0F1W1) to three climate-smart livestock practices (D1F1W1). Similarly, a livestock farmer adopting only one strategy for minimizing climate repercussions, such as diversification (D1F0W0), can gain 42.5 gm/day of protein by adopting all three (D1F1W1) CSLPs. Livestock farmers adopting a single feeding strategy (D0F1W0) as compared to adopters of all three strategies (D1F1W1), consumed 22.08 gm/day less per capita protein. The adoption of all climate-smart livestock practices (D1F1W1) by livestock farmers currently adopting only a weather-resistance strategy (D0F0W1) can add 33.87 gm/day to their per capita protein consumption.

#### 3.4.3. Adoption effect of CSLPs on Daily Calcium Intake

Table 8 depicts the average effect of the adoption of climate-smart livestock practices on the individual calcium consumption per day among study respondents. The livestock farmers adopting three practices jointly (D1F1WI) consumed an extra 68.30 mg/day of calcium than adopters of two practices (D1F1W0). Similarly, the livestock farmers with feed and weather resistance strategies (D0F1W1) on their farms consumed less calcium (72.62 mg/day) than the adopters of three strategies jointly (D1F1W1). These results indicate that diversification delivers more calcium intake when it is combined with feed and weather-resistance practices. The per capita calcium intake difference between adopters of three strategies jointly (D1F1W1) and single adopters (D1F0W0) is 266.67 mg/day. The results also indicate that diversification alone does not produce good results unless it is combined with the other two strategies viz. feed and weather-resistance practices, because the differences in per capita calcium consumption of adopters of a combination with two or three strategies and diversification in isolation are the largest as compared to the differences between feed and weather-resistance practices in isolation and the same combinations.

## 4. Discussion

The literature related to agriculture and climate change adaptation has recently started to investigate the connection between the adoption of climate-smart practices and the nutrition security of rural households. However, the current literature on the relationship between climate-smart agriculture practices and nutrition security ignores the interactions of CSLPs and nutrition security. Studies have begun to explore the factors that influence the adoption of CSLPs on farms. These studies report that the socioeconomic characteristics of rural households play a critical role in the choice of climate-smart measures to adopt on their farms [43,44,45,46,47,48,49].

A range of factors have been identified by this work that play a role in the adoption of a particular set of adaptation measures. The findings of this study on family size and adoption are not in line with the previous studies conducted by Ndamani and Watanabe [57], and the reason may be less involvement of young family members in livestock activities because the younger lot has less interest in livestock farming due to the lower return and physical labor required to perform livestock activities. More important than the total family size is the labor force linked with livestock activity and is positively associated with adoption. The gender of the household head presents mixed results, and different studies found different outcomes regarding the influence of gender on the adoption of CSLPs. It may be due to the difference in socio-cultural values between these societies. Belay et al. [58] found a positive relationship between male heads of household and the adoption of the climate-smart measures in this study. In contrast, Nhemachena and Hassan [59] reported a negative relationship between these two variables. Furthermore, human capital is one of the most influential factors affecting the adoption of CSLPs on livestock farms. Literature related to farming experience and adoption association also shows different outcomes, for example, [60,61] found a positive relationship between farming experience and the adoption of climate-smart practices. In contrast, Belay et al. [58] found a negative relationship between farming experience and the adoption of climate-smart practices, as in our study. The reason may be that experienced farmers are reluctant to take new measures and want to continue with the present practices learned from their ancestors. Similarly, Aryal et al. [46] reported a positive association between education and the adoption of climate-smart strategies on farms similar to this research finding. Findings on the impact of land holdings on the adoption of different CSLPs are in line with Ndamani and Watanabe [57] who report a positive impact of the former on the latter. Similarly, the study results related to animal inventory and the adoption of climate-smart strategies corroborate with Ali and Erenstein [62], who also reported a positive relationship between the adoption of climate-smart practices and animal inventory. Credit access and amount are also important indicators of adoption. As found in this study, Sadiq et al. [63] also noted a positive relationship between credit availability and the adoption of climate-smart practices at farms. The presence and use of various services such as livestock training, membership in farmer organizations, and weather information are considered important in the adoption of CSLPs, as they help disseminate information not only to participants or members but also to other livestock farmers through farmer-to-farmer extension. The results regarding the adoption of climate-smart measures and training are supported by the findings of Zakaria et al. [64], who reported a positive relationship between training and the adoption of climate-smart agricultural practices. Similarly, Maddison [65] found a positive association between adoption and weather information access. Similarly, the finding about the association of organizational membership with the adoption of climate-smart strategies is in line with [66].

The adoption of climate-smart practices significantly influences the food and nutrition security of households [43]. Diversification in combination with other strategies results in improved food nutrition, as also indicated by daily dietary intake [67]. Jones et al. [68] also found a strong association between dietary diversity and farm diversity in their studies. Farm diversity is also linked to providing more calories and protein consumption to rural households. In a similar vein, [31] reported that improved feeds with good breeds are helpful in decreasing poverty and can also contribute positively to households’ food nutrition and security. Similarly, weather-resistance climate measures such as shelter management and better drainage systems can improve animal productivity [20].

The results on food and nutrition security are also in line with previous studies conducted by Ali and Erenstein [62] and Teklewold et al. [51]. In this regard, [63] reported that farmers who adopt two strategies in farming have higher food security as compared to those who do not adopt any strategy. Similarly, farmers adopting three adaptation strategies are more food-secure as compared to farmers adopting only two strategies. Ali and Erenstein [62] suggested that rural households should be encouraged to adopt a higher number of strategies on farms. Teklewold et al. [51] reported that farmers who implemented three climate change strategies on their farms consumed more per capita calories and protein than farmers who implemented two climate strategies. Farmers who adopted two climate practices were found to be more food secure than farmers who used only one.

To conclude the discussion, this study is not without limitations. As in this survey, we relied on the recall ability of the farmers for the consumed quantities of agricultural commodities, which may lack accuracy. Moreover, it also ignores the usage of different bakery and fast-food items. Other researchers can build on this research by considering data on a larger number of food items.

## 5. Conclusions

This study can help the government achieve its food and nutrition sufficiency goals in a sustainable way as it investigates factors influencing different CSLPs and then creates a connection between adopted CSLPs and livestock households’ nutrition intake. Instead of just indicating who is food secure and who is not, this study estimated actual nutrition intake in the form of calories, protein, and calcium for livestock households. Livestock farmers were adopting diversification, feed, and weather-resistance strategies to minimize the impact of climate change on their farms.

The adoption of CSLPs on livestock farms was influenced by farmers’ demographics, human capital, physical and financial capital, and access to different services. Education, livestock inventory, livestock labor force, training, and total land were positively associated with the adoption of CSLP combinations involving two or more practices. The availability of credit for livestock farmers was also positively affecting the adoption of combinations involving feed strategies, whether in isolation or combined with other practices. Media usage for weather information was also positively associated with the adoption of all three practices jointly. On the other hand, framing experience was negatively associated with the adoption of all three CSLPs jointly.

The empirical results of this study confirmed that the adoption of a higher number of CSLPs at livestock farms is associated with higher consumption of per capita dietary intakes. Moreover, livestock farmers adopting a single practice on their farms consumed a lower per capita dietary intake as compared to livestock farmers adopting two or more practices together on their farms. Similarly, livestock farmers adopting two or more CSLPs jointly consumed more diversified food compared to livestock farmers who adopted a single CSLP at farms.

To sum up, the study has important policy implications for developing countries, particularly Pakistan. To begin with, the study highlights the nutritional value of using a greater number of CSLPs on livestock farms. Secondly, the results highlight the importance of access to different services such as credit, training, and media in the adoption of CSLPs at the farm level. Therefore, government agencies in developing nations should focus on two aspects to increase the adoption of CSLPs for improving the food and nutrition security of rural households: (i) increasing the awareness and knowledge about the benefits of adopting a greater number of CSLPs at farms; and (ii) improving training services’ access to more livestock farmers and should organize more frequent training programs to enhance adoption at the farm level.

## Figures and Tables

**Table 1:** Different combinations of CSLPs and their adoption status.

Choice (*j*)	Combinations	Components of Combinations			
		Diversification Practices	Feeding Practices	Weather-Resistance Practices	Adoption
1	D1F1W1	Yes	Yes	Yes	0.35
2	D1F1W0	Yes	Yes	-	0.39
3	D1F0W1	Yes	-	Yes	0.43
4	D0F1W1	-	Yes	Yes	0.27
5	D1F0W0	Yes	-	-	0.10
6	D0F1W0	-	Yes	-	0.09
7	D0F0W1	-	-	Yes	0.13
8	D0F0H0	-	-	-	-

“-” sign indicates non-adoption of a combination of CSLPs.

**Table 2:** Description of explanatory variables.

Variable	Description	Expected Contribution
Demographics		
Age	Age of household in years	±
Family size	Total family persons in household	±
Gender of household head	Dummy, 1 if male household head, otherwise 0	±
Human capital		
Education	Education of household in years	+
Labor force	Adult persons available for livestock activities	+
Livestock experience	Livestock experience of household head in years	±
University education	Dummy, 1 if anyone from house have university level education, otherwise 0	+
Financial and physical capitals		
Total land	Total operated land in acres	+
Off farm income	Dummy, 1 if off-farm income source, otherwise 0	+
Animal inventory	Livestock units	+
Credit access	Dummy, 1 If credit facility availed, otherwise 0	+
Access to services		
Market distance	Market distance from home in kilometers	−
Veterinary hospital distance	Veterinary hospital distance from farm in kilometers	+
Veterinary doctor availability	Dummy, 1 if veterinary doctor available easily, otherwise 0	+
Training workshop	Dummy, 1 if participated in any livestock training/workshop otherwise 0	+
Membership of organization	Dummy, 1 if member of farmer organization, otherwise 0	+
Media access	Dummy, 1 if TV or radio is used for weather information, otherwise 0	+

The “+” sign denotes a predicted positive effect, the “−” sign indicates a predicted negative effect, and the “±” symbol denotes a predicted both positive and negative influence of relevant variables on CSLPs adoption.

**Table 3:** Characteristics of different adopter groups of CSLPs.

Variables	D1F1W1	D1F1W0	D1FOW1	D0F1W1	D1F0W0	D0F1W0	D0F0W1	Over All
	Mean	Mean	Mean	Mean	Mean	Mean	Mean	Mean
Demographics								
Age (year)	47.03 ^a^ (4.49)	48.51 ^a^ (4.90)	52.11 ^a^ (5.74)	50.19 ^a^ (5.14)	51.57 ^a^ (5.68)	52.60 ^a^ (4.10)	50.90 ^a^ (5.30)	50.93 (5.13)
Family size (persons)	7.76 ^a^ (1.09)	8.49 ^a^ (1.96)	8.40 ^a^ (1.25)	7.96 ^a^ (1.84)	7.85 ^a^ (1.90)	8.20 ^a^ (1.76)	8.37 ^a^ (1.93)	8.19 (1.91)
Gender of household head (male = 1)	0.91 ^a^ (0.28)	0.93^a^ (0.23)	0.90 ^a^ (0.30)	0.92 ^a^ (0.23)	0.95 ^a^ (0.22)	0.96 ^a^ (0.22)	1.00 ^a^ (0.00)	0.94 (0.16)
Human capital								
Education (years)	8.23 ^a^ (1.12)	8.0 7^a^ (1.55)	7.95 ^a^ (0.94)	7.08 ^a^ (1.44)	6.79 ^a^ (1.78)	7.10 ^a^ (1.90)	7.40 ^a^ (2.10)	7.90 (1.71)
Labor force (persons)	2.44 ^a^ (0.70)	2.10 ^a^ (0.89)	1.90 ^b^ (0.45)	1.73 ^b^ (0.37)	2.10 ^a^ (0.50)	1.65 ^b^ (0.55)	1.40 ^e^ (0.43)	1.83 (0.57)
University education (yes = 1)	0.33 ^a^ (0.49)	0.46 ^b^ (0.48)	0.25 ^a,c,d^ (0.44)	0.19 ^c,d,e^ (0.42)	0.21 ^d^ (0.41)	0.11 ^e,f^ (0.33)	0.09 ^f^ (0.31)	0.31 (0.47)
Physical and financial capital								
Total land (acres)	8.21 ^a^ (1.06)	6.69 ^b,c^ (1.85)	6.25 ^b^ (1.72)	6.76 ^c^ (0.69)	5.85 ^d,b^ (1.20)	5.40 ^e,d^ (1.52)	4.70 ^f^ (1.30)	6.17 (1.35)
Livestock experience (years)	13.12 ^a^ (3.66)	17.79 ^b,d^ (4.68)	17.25 ^c,b^ (4.61)	18.15 ^d,e^ (4.15)	18.34 ^e,d^ (4.10)	20.65 ^f^ (4.90)	17.90 ^g,b,c,d^ (5.30)	17.43 (4.23)
Animal inventory (livestock unit)	8.10 ^a^ (1.70)	6.55 ^b^ (1.20)	4.60 ^c^ (0.65)	5.89 ^d,b^ (0.60)	5.10 ^e,d^ (1.00)	3.56 ^f^ (0.79)	4.32 ^g,f,c^ (1.90)	5.45 (1.14)
Off-farm income (yes = 1)	0.97 ^a^ (0.17)	0.68 ^b^ (0.42)	0.73 ^c,b^ (0.48)	0.62 ^d,b,c^ (0.50)	0.52 ^e,d^ (0.51)	0.23 ^f^ (0.43)	0.13 ^g^ (0.34	0.55 (0.50)
Credit access (yes = 1)	0.51 ^a^ (0.50)	0.49 ^a^ (0.46)	0.44 ^a^ (0.50)	0.46 ^a^ (0.51)	0.21 ^b^ (0.41)	0.11 ^c^ (0.33)	0.10 ^c^ (0.32)	0.36 (0.49)
Access to services								
Market distance (kilometer)	6.03 ^a,c^ (1.73)	4.44 ^b,e,f^ (1.31)	5.89 ^c,a,g^ (1.95)	5.12 ^d,g^ (1.52)	4.10 ^e,b^ (1.80)	4.68 ^f,e,b^ (1.23)	5.54 ^g,c,d^ (1.90)	5.10 (1.75)
Veterinary hospital distance (kilometer)	4.97 ^a^ (0.87)	2.99 ^b^ (1.06)	5.81 ^a^ (1.46)	5.46 ^a^ (1.56)	4.96 ^a^ (1.14)	5.32 ^a^ (1.20)	4.29 ^a^ (1.30)	4.67 (1.60)
Veterinary doctor facility (yes = 1)	0.65 ^a^ (0.49)	0.41 ^b,d,c,e,g^ (0.49)	0.39 ^c,b,d,e,g^ (0.49)	0.42 ^d,c,b,e,g^ (0.50)	0.39 ^e,b,c,d,g^ (0.49)	0.20 ^f^ (0.40)	0.38 ^g,b,c,d,e^ (0.48)	0.42 (0.49)
Training/workshop (yes = 1)	0.46 ^a^ (0.50)	0.19 ^b^ (0.39)	0.17 ^b^ (0.39)	0.14 ^b^ (0.35)	0.15 ^b^ (0.37)	0.05 ^b^ (0.22)	0.10 ^b^ (0.32)	0.17 (0.38)
Organization membership (yes = 1)	0.42 ^a^ (0.53)	0.20 ^b^ (0.37)	0.12 ^b^ (0.33)	0.06 ^d,c^ (0.25)	0.15 ^b^ (0.37)	0.11 ^b,c^ (0.33)	0.05 ^d,c^ (0.22)	0.14 (0.42)
Media access (yes = 1)	0.32 ^a^ (0.47)	0.42 ^b^ (0.50)	0.52 ^c^ (0.51)	0.47 ^b,c^ (0.52)	0.22 ^d^ (0.42)	0.22 ^d^ (0.42)	0.20 ^d^ (0.46)	0.35 (0.47)

S.D. stands for standard deviation here. Unlike superscripts (a–g) along the row show a significant difference. The values in the parenthesis are standard deviations.

**Table 4:** Nutritional status of rural households.

Nutrition Indicator	Unit	Nutrition Status
Calorie	kcal/day/adult	2413.19 (463.95)
Protein	mg/day/adult	69.90 (18.11)
Calcium	mg/day/adult	657.70 (204.78)
Nutrition diversity based on calorie share		0.67 (0.11)
Nutrition diversity based on protein share		0.70 (0.07)
Nutrition diversity based on calcium share		0.65 (0.13)

The values in the parentheses are standard deviations.

**Table 5:** Factors affecting the adoption of different CSLP combinations.

Variables	D1F1S1	D1F1W0	D1F0W1	D0F1W1	D1F0W0	D0F1W0	D0F0W1
	Coef.	Coef.	Coef.	Coef.	Coef.	Coef.	Coef.
Demographics							
Age	0.03 (0.02)	−0.03 *** (0.02)	−0.07 ** (0.04)	−0.13 * (0.03)	−0.02 (0.02)	−0.01 (0.02)	−0.05 ** (0.02)
Family size	−0.03 (0.08)	−0.18 ** (0.08)	−0.33 ** (0.15)	−0.10 (0.09)	−0.24 *** (0.13)	−0.08 (0.12)	0.04 (0.11)
Gender of household head	0.97 ** (0.51)	0.25 (0.43)	0.912 (0.73)	0.97 *** (0.56)	0.20 (0.51)	1.30 * (0.48)	−0.81 (0.59)
Human capital							
Education	0.24 * (0.05)	0.15 ** (0.06)	0.14 *** (0.08)	0.13 ** (0.05)	(0.21 (0.18)	0.09 (0.18)	0.15 (0.15)
Livestock experience	−0.05 *** (0.02	0.01 (0.02)	−0.08 ** (0.03)	−0.74 * (0.03)	−0.00 (0.02)	−0.05 ** (0.02)	0.001 (0.02)
University education	1.23 * (0.35)	−0.09 (0.23)	0.34 (0.64)	0.33 (0.33)	0.70 *** (0.43)	0.41 (0.39)	0.02 (0.33)
Physical and financial capital							
Livestock labor force	0.46 * (0.13)	0.17 *** (0.10)	0.44 ** (0.19)	0.53 * (0.13)	0.14 (0.16)	0.013 (0.15)	−0.12 (0.15)
Total land	0.08 ** (0.04)	0.08 *** (0.04)	0.12 *** (0.06)	0.11 *** (0.06)	0.14 ** (0.05)	0.18 (0.97)	0.07 (0.05)
Animal inventory	0.23 * (0.07)	0.25 * (0.07)	0.36 * (0.13)	−0.03 (0.69)	0.03 (0.07)	0.12 *** (0.07)	0.06 (0.05)
Off-farm income	1.50 * (0.34)	0.38 (0.34)	0.37 (0.43)	0.98 ** (0.44)	1.11 ** (0.44)	1.07 ** (0.45)	1.0 5** (0.45)
Credit access	0.07 (0.24)	0.15 ** (0.05)	0.19 (0.30)	0.55 ** (0.22)	−0.27 (0.22)	0.55 ** (0.23)	0.106 (0.21)
Access to services							
Market distance	−0.16 (0.24)	−0.68 * (0.06)	0.12 (0.30)	0.13 (0.22)	−0.74 * (0.24)	−0.30 (0.24)	0.34 (0.24)
Training/workshop	0.79 * (0.17)	0.66 * (0.16)	0.63 ** (0.32)	0.55 ** (0.23)	0.32 (0.24)	0.20 (0.25)	0.76 * (0.23)
Membership of organization	0.99** (0.39)	0.37 (0.37)	1.11 (0.39)	0.39 (0.34)	0.27 (0.35)	0.36 (0.33)	−0.11 (0.31)
Media access	0.26 ** (0.10)	0.68 * (0.06)	0.12 (0.30)	0.13 (0.22)	0.74 * (0.24)	0.30 (0.24)	0.16 (0.24)
Constant	−5.09 * (1.93)	3.01 ** (1.50)	3.81 (2.49)	8.86 (1.94	5.02 * (1.74)	3.52 (1.65)	4.78 * (1.67)
Log likelihood = −446.00, Wald chi2 = 270, Prob > chi2 = 0.000							

*, **, and *** indicate significant difference at 1%, 5%, and 10%, respectively. S.E. stands for standard error here. The values in the parentheses are standard errors.

**Table 6:** Average effect of adopted CSLPs on calorie consumption per adult equivalent (kcal/day).

Sample	Outcome	Adoption Status		Average Difference
		Full Adoption (*j* = 1)	Partial Adoption (*j* = 2)	
(a) Full adopter	*E*(*Y_j_*/*I* = 1)	2883.6 (40.9)	2590.78 (23.3)	292.81 (42.24) *
		Full adoption (*j* = 1)	Partial adoption (*j* = 3)	
(b) Full adopter	*E*(*Y_j_*/*I* = 1)	2883.6 (40.9)	2535.32 (30.9)	348.28 (41.04) *
		Full adoption (*j* = 1)	Partial adoption (*j* = 4)	
(c) Full adopter	*E*(*Y_j_*/*I* = 1)	2883.6 (40.9)	2389.44 (34.67)	494.16 (50.2)*
		Multiple adoption (*j* = 1, 2, 3)	Single adoption (*j* = 5)	
(d) Multiple adopter	*E*(*Y_j_*/*I* = 1)	2883.6 (40.9)	2215.78 (94.32)	667.82 (103.83) *
	*E*(*Y_j_*/*I* = 2)	2549.3 (27.39)	2105.39 (89.30)	443.90 (91.33) *
	*E*(*Y_j_*/*I* = 3)	2477.72 (32.30)	2139.83 (85.30)	337.89 (97.53) *
		Multiple adoption (*j* = 1, 3, 4)	Single adoption (*j* = 6)	
(e) Multiple adopter	*E*(*Y_j_*/*I* = 1)	2883.6 (40.9)	2383.4 (98.45)	500.2 (110.78) *
	*E*(*Y_j_*/*I* = 3)	2477.72 (32.30)	2240.23 (87.32)	237.48 (102.43) **
	*E*(*Y_j_*/*I* = 4)	2280.49 (29.30)	2230.00 (92.32)	50.49 (100.20)
		Multiple adoption (*j* = 1, 2, 4)	Single adoption (*j* = 7)	
(f) Multiple adopter	*E*(*Y_j_*/*I* = 1)	2883.6 (40.9)	2325.3 (101.3)	558.3(106.82) *
	*E*(*Y_j_*/*I* = 2)	2549.3 (27.39)	2380.96 (93.34)	168.34 (98.19)
	*E*(*Y_j_*/*I* = 4)	2280.49 (29.30)	2260.24 (90.67)	20.25 (96.50)

* and ** indicate significant difference at 1% and 5%, respectively. The values in the parentheses are standard errors.

**Table 7:** Average effect of adopted practices on protein consumption per adult equivalent (gm/day).

Sample	Outcome	Adoption Status		Average Difference
		Full Adoption (*j* = 1)	Partial Adoption (*j* = 2)	
(a) Full adopter	*E*(*Y_j_*/*I* = 1)	92.87 (0.85)	85.09 (2.79)	07.77 (3.17) **
		Full adoption (*j* = 1)	Partial adoption (*j* = 3)	
(b) Full adopter	*E*(*Y_j_*/*I* = 1)	92.87 (0.85)	81.69 (2.13)	19.18 (2.41) *
		Full adoption (*j* = 1)	Partial adoption (*j* = 4)	
(c) Full adopter	*E*(*Y_j_*/*I* = 1)	92.87 (0.85)	71.18 (3.2)	21.68 (3.43) *
		Multiple adoption (*j* = 1, 2, 3)	Single adoption (*j* = 5)	
(d) Multiple adopter	*E*(*Y_j_*/*I* = 1)	92.87 (0.85)	50.37 (2.59)	42.5(2.70) *
	*E*(*Y_j_*/*I* = 2)	91.75 (1.12)	55.84 (2.90)	35.41(3.77) *
	*E*(*Y_j_*/*I* = 3)	79.46 (0.77)	48.49 (3.13)	30.97(3.28) *
		Multiple adoption (*j* = 1, 3, 4)	Single adoption (*j* = 6)	
(e) Multiple adopter	*E*(*Y_j_*/*I* = 1)	92.87 (0.85)	70.79 (7.20)	22.08 (7.64) *
	*E*(*Y_j_*/*I* = 3)	79.46 (0.77)	70.57 (7.45)	8.89 (7.51)
	*E*(*Y_j_*/*I* = 4)	65.33 (0.90)	61.24 (8.55)	4.59(8.70)
		Multiple adoption (*j* = 1, 2, 4)	Single adoption (*j* = 7)	
(f) Multiple adopter	*E*(*Y_j_*/*I* = 1)	92.87 (0.85)	57 (5.10)	33.87(5.19) *
	*E*(*Y_j_*/*I* = 2)	91.75 (1.12)	62.57 (6.90)	29.18 (7.51) *
	*E*(*Y_j_*/*I* = 4)	65.33 (0.90)	53.38 (4.64)	11.95(4.83) **

* and ** indicate significant difference at 1% and 5%, respectively. The values in the parentheses are standard errors.

**Table 8:** Average effect of adopted practices on calcium consumption per adult equivalent (mg/day).

Sample	Outcome	Adoption Status		Average Difference
		Full Adoption (*j* = 1)	Partial Adoption (*j* = 2)	
(a) Full adopter	*E*(*Y_j_*/*I* = 1)	757.62 (15.54)	689.6 (24.5)	68.30 (30.87) **
		Full adoption (*j* = 1)	Partial adoption (*j* = 3)	
(b) Full adopter	*E*(*Y_j_*/*I* = 1)	757.62 (15.54)	798.84 (32.2)	(−41.62) (37.11)
		Full adoption (*j* = 1)	Partial adoption K = 4	
(c) Full adopter	*E*(*Y_j_*/*I* = 1)	757.62 (15.54)	685.27 (27.43)	72.62 (38.97) ***
		Multiple adoption (*j* = 1, 2, 3)	Single adoption (*j* = 5)	
(d) Multiple adopter	*E*(*Y_j_*/*I* = 1)	757.62 (15.54)	490.95 (34.23)	266.67 *(39.22)
	*E*(*Y_j_*/*I* = 2)	737.3 (27.26)	500.745 (49.48)	236.56 (54.49) *
	*E*(*Y_j_*/*I* = 3)	786.22 (32.56)	544.22 (39.23)	242.00 (49.59) *
		Multiple adoption (*j* = 1, 3, 4)	Single adoption (*j* = 6)	
(e) Multiple adopter	*E*(*Y_j_*/*I* = 1)	757.62 (15.54)	681.35 (25.24)	76.27 (27.20) **
	*E*(*Y_j_*/*I* = 3)	786.22 (32.56)	651.08 (27.82)	135.14 (28.86) *
	*E*(*Y_j_*/*I* = 4)	662.97 (23.3)	627.00 (29.00)	35.97 (30.95)
		Multiple adoption (*j* = 1, 2, 4)	Single adoption (*j* = 7)	
(f) Multiple adopter	*E*(*Y_j_*/*I* = 1)	757.62 (15.54)	626.58 (30.7)	131.62 (36.48) *
	*E*(*Y_j_*/*I* = 2)	737.3 (27.26)	588.15 (38.3)	149.15(48.10) *
	*E*(*Y_j_*/*I* = 4)	662.97 (23.3)	550.7 (47.32)	112.26 (52.73) **

*, **, and *** indicate significant difference at 1%, 5%, and 10%, respectively. The values in the parentheses are standard errors.

## Data Availability

Data is contained with the article.
